# Pathogenic marine microbes influence the effects of climate change on a commercially important tropical bivalve

**DOI:** 10.1038/srep32413

**Published:** 2016-08-31

**Authors:** Lucy M. Turner, Christian Alsterberg, Andrew D. Turner, S. K. Girisha, Ashwin Rai, Jonathan N. Havenhand, M. N. Venugopal, Indrani Karunasagar, Anna Godhe

**Affiliations:** 1 Department of Marine Sciences, University of Gothenburg, Box 461, SE 405 30 Göteborg, Sweden; 2 Centre for Environment, Fisheries and Aquaculture Science (CEFAS), Barrack Road, The Nothe, Weymouth, Dorset, DT4 8UB, United Kingdom; 3 Department of Fishery Microbiology, Karnataka Veterinary Animal and Fisheries Sciences University, College of Fisheries, Mangalore, 575002, India; 4 UNESCO-MIRCEN for Medical and Marine Biotechnology, Nitte University Centre for Science Education and Research (NUCSER), Nitte University, Mangalore 575018, India

## Abstract

There is growing evidence that climate change will increase the prevalence of toxic algae and harmful bacteria, which can accumulate in marine bivalves. However, we know little about any possible interactions between exposure to these microorganisms and the effects of climate change on bivalve health, or about how this may affect the bivalve toxin-pathogen load. In mesocosm experiments, mussels, *Perna viridis,* were subjected to simulated climate change (warming and/or hyposalinity) and exposed to harmful bacteria and/or toxin-producing dinoflagellates. We found significant interactions between climate change and these microbes on metabolic and/or immunobiological function and toxin-pathogen load in mussels. Surprisingly, however, these effects were virtually eliminated when mussels were exposed to both harmful microorganisms simultaneously. This study is the first to examine the effects of climate change on determining mussel toxin-pathogen load in an ecologically relevant, multi-trophic context. The results may have considerable implications for seafood safety.

Multi-model projections of future global climate suggest that sea surface temperature (SST) and precipitation maxima will increase^1^. Tropical areas, with monsoon driven weather patterns, such as SW India are projected to experience some of the most extreme rainfall events^2^, with increased freshwater run-off likely to result in decreased sea surface salinity (SSS) in coastal waters^3^,^4^. Together these factors are known to induce structural changes in the oceanic microbial community, in terms of both species abundance and composition^5^,^6^,^7^, and are thought to play a part in the apparent increase of Harmful Algal Blooms (HABs) worldwide^8^. Documented associations between environmental changes and prevalence of pathogenic bacteria also indicate elevated infection risk in correspondence with projected warming trends^9^,^10^.

Marine microbes favoured by climate change, such as pathogenic bacteria and bloom-forming, toxin-producing phytoplankton, may accumulate in commercially important filter feeders, subsequently passing to higher trophic levels^11^,^12^,^13^, causing a severe risk to human health^14^,^15^. These impacts are often magnified in tropical regions, where a significant proportion of the coastal population depend on seafood as an economic and/or protein resource^16^. Therefore, both large and small scale outbreaks of *Vibrio* and shellfish toxin contamination (e.g. paralytic shellfish toxin (PST)), for example, can have significant economic as well as social consequences^17^.

Climate change induced warming and hyposalinity are likewise known to have significant detrimental effects on the metabolic and immunobiological performance of marine bivalves^18^,^19^. Previous work with *Perna viridis* has demonstrated that exposure to sub-lethal salinities can result in detrimental changes in behaviour (e.g. reductions in byssal thread production and valve closure^20^), immune system parameters (e.g. increased haemocyte mortality and decreased phagocytosis, esterase, reactive oxygen species, lysosome content and total haemocyte counts^21^) and increased respiration rate (MO_2_)^22^. Elevated temperatures have been reported to reduce immune response markers^23^ and increase MO_2_^24^,^25^ and heart rate^26^.

Similar behavioural and physiological responses (e.g. changes to filtration rate, valve closure, growth rate, byssus thread production and MO_2_) have been recorded when bivalves were exposed to toxic dinoflagellates, with the strength of these responses often varying with bivalve species, population and geographical location^27^,^28^. In previous experiments with *P. viridis* behavioural responses, such as upregulation of ingestion avoidance behaviour, has been shown to result in decreased food absorption, leading to a decrease in energy supply^29^,^30^. Bacterial loading in bivalves is dictated by the bactericidal activity of the haemolymph^31^,^32^. However very high bacterial loads, possibly where the immune system is already compromised, can induce further significant changes to the immunobiological response and also adversely affect metabolic regulation^33^,^34^,^35^. Despite these effects on bivalve physiology being relatively well characterised when measured separately, we still know little about possible interactions between the microorganisms on which bivalves feed and the effects of climate change on the physiological processes underpinning the health of marine shellfish. Equally, therefore, we know virtually nothing about how these interactions may affect the toxin-pathogen load of commercially important seafood^36^.

In this study, we investigated how the toxin-pathogen load and the mechanisms that underpin the physiological health of the tropical, commercially important, green mussel *P. viridis* are affected by climate change induced warming and/or hyposalinity and exposure to the pathogenic bacteria *Vibrio parahaemolyticus* (‘*Vibrio*’) and/or the PST producing dinoflagellate *Alexandrium minutum* (‘*Alexandrium*’). We especially focussed on the relative importance of direct and indirect effects of these on the physiological pathways which ultimately affect mussel health and overall toxin-pathogen load, for which we used Structural Equation Modelling (SEM) (see Methods). In the area of SW India where we performed our experiment *P. viridis* experiences seasonal spikes of high temperatures and/or lowered salinity which approach the limits of its known tolerance range^25^,^37^. However, here it is unusual for mussels to experience high temperatures and/or lowered salinity concurrently at the levels used in our experiment. Mussels were maintained in mesocosms under either ambient or projected climate change conditions (warming and hyposalinity), with or without dinoflagellates and *Vibrio*. Total toxin-pathogen load (PST and *Vibrio* concentration) was subsequently quantified and immunobiological and metabolic aspects of mussel physiological function were examined. We hypothesised that: *i*) the effects of warming and hyposalinity would have detrimental effects on metabolic and immunobiological function in *P. viridis* leading to an increase in mussel toxin-pathogen load; *ii*) these effects would be magnified when mussels were also exposed to pathogenic or toxic microorganisms and; *iii*) any indirect effects of warming and hyposalinity and exposure to the microbes on the physiological mechanisms would be at least as strong as the direct effects.

## Results

### General Linear Model (GLM) Analyses

**Figure 1:**
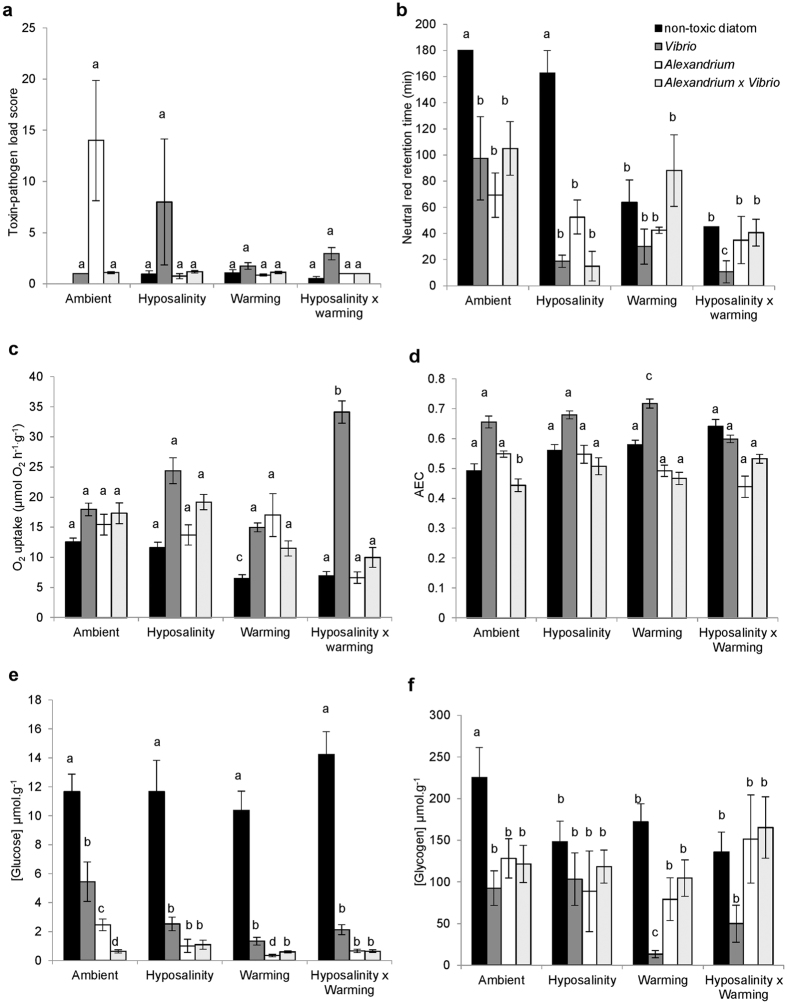
The interactive effects of microorganism exposure and simulated climate change effects on some aspects of physiological function in *P. viridis*. The (**a**) toxin-pathogen load score, (**b**) immnobiological status (lysosomal membrane stability), (**c**) oxygen consumption (**d**) adenylate energy charge (AEC), (**e**) glucose (μmol g^−1^), and (**f**) glycogen (μmol g^−1^) of *Perna viridis* following 14 day’s exposure to the non-toxin producing diatom *Thalassiosira weissflogii*, the toxin producing dinoflagellate *Alexandrium minutum* and/or the pathogenic bacteria *Vibrio parahaemolyticus* (fed together with *T. weissflogii*) under differing climate change conditions. Within each graph, different letters indicate means that are significantly different from each other (*P* < 0.05), but see also Tables 1, 2 and 3. Data are means ± SEM. Samples sizes (n = 16) but see Supplementary Table 2.

There were no significant effects of simulated climate change or microorganism exposure, but there was a significant interaction effect on overall mussel toxin-pathogen load (Fig. 1a, Table 1). Regarding immune function, there was a significant interaction between the effects of warming + hyposalinity on immunobiologcal status (measured as lysosomal membrane stability via neutral red retention; *P* < 0.001, Table 2) with immune function significantly lower in mussels exposed to warming + hyposalinity. Immunobiological status was also significantly negatively affected by all three toxin-pathogen microorganism exposures (*Vibrio, P* < 0.001, *Alexandrium, P* = 0.001 and *Vibrio* + *Alexandrium, P* = 0.038) when compared to those mussels fed the non-toxic diatom (Fig. 1b, Table 3). However, when *Alexandrium* + *Vibrio* were fed together and mussels were exposed to warming + hyposalinity, the presence of *Alexandrium* had a mediating effect: immunity (as neutral red retention) was significantly higher than that in mussels fed *Vibrio* only (Fig. 1b). This mediating effect of *Alexandrium* persisted across several metabolic parameters investigated, including oxygen consumption and ATP concentration (Fig. 1d and Supplementary Fig. 1d). Mussels fed *Alexandrium* + *Vibrio* always displayed values closer to those of control animals than those fed *Vibrio* only. There was no significant effect of microorganism exposure (*P* = 0.323) or climate change (*P* = 0.303) on gill Na^+^/K^+^-ATPase activity (Supplementary Fig. 1c, Table 1). However, microorganism exposure had a significant negative effect on both glucose and glycogen concentration (Fig. 1e–f), but no effect of climate change was observed (Table 1). Power analysis (the likelihood that our tests would have detected a biologically meaningful effect had it existed) showed that the GLM tests had >80% power.

### Structural Equation Modelling (SEM) Analyses

**Figure 2:**
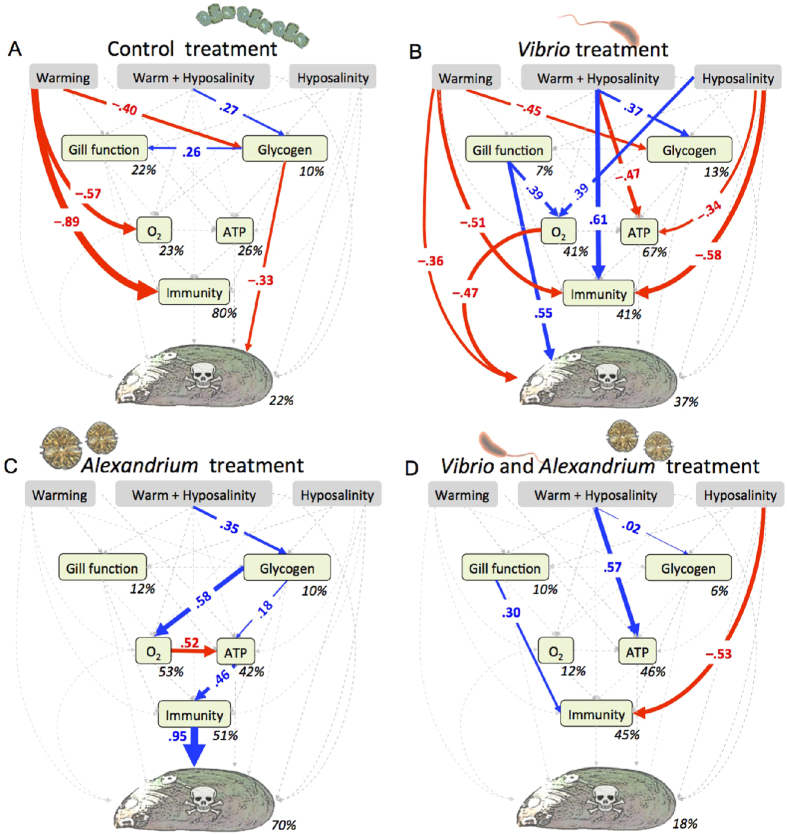
Path diagrams showing the interactive effects of microorganism exposure and simulated climate change effects on toxin-pathogen load in *P. viridis*. Path diagrams showing how experimental warming, hyposalinity, and the interaction between warming and hyposalinity affect the gill function, glycogen, oxygen consumption (O_2_), ATP, immunity and toxin-pathogen load. Path diagrams represent (**A**) mussels feed with non-toxic diatoms (*Thalassiosira weissflogii*), (**B**) mussels exposed to *Vibrio*, (**C**) mussels exposed to *Alexandrium* and, (**D**) mussels exposed to *Vibrio* + *Alexandrium*. Solid paths (blue and red) are (positively and negatively) statistically significant (*P* < 0.05) whereas the dashed grey lines are not. Note that a positive effect on ‘toxicity’ results in an increased toxicity level within the mussels, whereas a negative effect results in a decreased toxicity level. At each significant path the standardised coefficients are represented and interpreted as follows: If, for example, temperature goes up by 1 SD during *Vibrio* exposure, the immunity of mussels goes down by 0.51 SD. Percentages indicate the variance explained by the model. Samples sizes (n = 16) but see Supplementary Table 2.

Structural Equation Modelling (SEM) allows the partitioning of net effects into direct and indirect effects, and thus an estimation of their relative importance. Our use of SEM enabled us to disentangle any direct and indirect effects of simulated climate change and microorganism exposure on the physiological pathways which ultimately affect mussel health and overall toxin-pathogen load. Individual and multigroup SEM models were not statistically different from the observed data (Table 4). SEM analyses revealed strong direct and indirect effects of warming and hyposalinity on mussel metabolic and immunobiological function and toxin-pathogen load (Fig. 2). The physiological response of the mussels differed with feeding regime (non-toxic diatoms, *Vibrio, Alexandrium,* or *Vibrio + Alexandrium*). The most complex network of effects was observed in the presence of *Vibrio* (Fig. 2B), where exposure to warming, hyposalinity, and warming + hyposalinity all had significant effects on mussel metabolic and immunobiological parameters, which led to an increase in mussel toxin-pathogen load (−0.28) compared to mussels fed non-toxic diatoms (−0.33). In mussels exposed to *Alexandrium,* warming + hyposalinity had significant effects on metabolic and immunobiological function which mediated the greatest increase in mussel toxin-pathogen load (0.95) (Fig. 2C). Unexpectedly, when mussels were fed *Alexandrium* + *Vibrio* almost all direct and indirect climate change effects on mussel physiology were non-significant and there were no significant direct or indirect effects on the toxin-pathogen load (Fig. 2D). Furthermore, in all analyses, the direct and indirect effects of warming and hyposalinity in many cases cancelled each other out, resulting in low overall effects on the toxin-pathogen load. For example, the direct negative effect of warming on the toxin-pathogen load in the mussels exposed to *Vibrio* (0.36) was reduced to 0.08 when the positive indirect effects (0.28) were taken into consideration (Supplementary Table 4b). This most likely explains why the ANOVA analysis of the toxin-pathogen load found no overall significant effects of warming or hyposalinity on the total toxin-pathogen load (Fig. 1a).

## Discussion

This study demonstrates that organismal responses to climate change can be strongly modified by interactions within the food chain. More specifically, we show that exposure to *Vibrio* amplified statistically significant direct negative effects of warming and hyposalinity on mussel metabolic and immunobiological functioning, ultimately leading to an increased toxin-pathogen load (Fig. 2B). Toxin-pathogen load was even higher, and there was higher mortality (Supplementary Table 1) when mussels were exposed to the toxic dinoflagellate *Alexandrium –* a result that was due to the indirect effects of both warming and hyposalinity on metabolic and immunobiological pathways (Fig. 2C). However, when mussels were fed *Alexandrium + Vibrio*, nearly all negative effects of warming and hyposalinity on physiological functions dissipated and there were no direct or indirect effects on the toxin-pathogen load (Fig. 2D).

Mussels exposed to warming had reduced glycogen concentrations, oxygen consumption and immune function (Fig. 2A). Warming also indirectly affected the toxin-pathogen load via negative effects on glycogen concentration, whereas no indirect single effects of hyposalinity were observed. This supports the assertion that warming induces changes in glycogen concentration and depletes carbohydrate reserves due to increased metabolic challenges^19^ which may also result in a cost to other physiological systems, including immune function^38^. Together this can influence mussel toxin-pathogen load^23^.

In our experiment, when heat-stressed mussels were fed pathogenic microorganisms, the physiological response to warming and hyposalinity changed. Significant negative effects of warming and hyposalinity on metabolic and immunobiological function were only observed when mussels were exposed to the pathogenic bacteria *Vibro,* leading to an increase in overall toxin-pathogen load (Fig. 2B). This was due to a direct effect of warming combined with an indirect effect of hyposalinity via upregulation of oxygen consumption and an increase in gill function. However, there were also direct negative effects on immune system function and metabolism indicated by a decline in glycogen and ATP concentrations. Collectively, these responses indicate that the mussels entered a reduced energy state, driven by the need to meet the twin challenges of maintaining immune system function and upregulating the metabolic response^23^,^39^,^40^. When exposed to hyposaline conditions, any upregulation of homeostatic ion-osmotic mechanisms will also require energy in the form of ATP, leading to increased oxygen demand^22^. Indeed, we also observed this effect of hyposalinity on oxygen consumption, which significantly increased under hyposaline conditions. The upregulation of mussel filtration rate has also been reported to be stimulated by warming in *P. viridis,* reaching an optimum at 30 °C^25^ which suggests that temperature could have a direct effect on behaviour that could in turn play a part in regulating the toxin-pathogen load. At temperatures beyond 30 °C mussel filtration rate would likely decline and cause a concomitant decline in ingestion rate of pathogens. This could eventually lead to a decreased toxin-pathogen load - a result that we observed.

The largest increase in the toxin-pathogen load was seen in mussels exposed to warming + hyposalinity while simultaneously being fed the toxin producing dinoflagellate *Alexandrium* (Fig. 2C). Those mussels responded by upregulating their metabolic machinery; increasing oxygen consumption with both direct and indirect effects on the glycogen pool and ATP concentrations. Glycogenesis increased as did glycogenolysis. Glycolysis was also likely upregulated fuelling ATP production. Although this metabolic upregulation had a positive effect on immunity, this was not enough to prevent a large warming + hyposalinity-induced increase in the overall toxin-pathogen load, which would likely be detrimental in the long run. Prolonged challenges by PST toxins on mussels are known to cause widespread inflammatory responses and also have damaging effects on the immune system, often leading to further pathological changes and parasitic infection^41^.

Surprisingly, when we fed the mussels *Alexandrium* + *Vibrio*, the presence of *Alexandrium* counteracted the negative effects of *Vibrio* on immunometabolic functions. Almost all the direct and indirect effects of warming and hyposalinity on the physiology of *Vibrio* exposed mussels were non-significant when mussels were fed *Alexandrium* + *Vibrio* (Fig. 2D). This pattern also applied to toxin-pathogen load. Pathogenic marine bacteria such as *Vibrio* are known to have algicidal effects on PST-producing dinoflagellates such as *Alexandrium*^42^,^43^,^44^, and have therefore been suggested for use in the control of HABs^45^. However, our results (Fig. 2D) indicate that toxic dinoflagellates may also be able to ameliorate the negative effects of pathogenic marine bacteria. A similar interaction has recently been identified in clams, in which *Alexandrium tamarense* reduced the effects of *Vibrio tapetis*, the cause of brown ring disease^46^. Several interactive mechanisms between *Vibrio* and *Alexandrium* can be identified as potential explanations for our results. First *Alexandrium* may be directly toxic to *Vibrio*, and secondly, exposure to *Alexandrium* could have stimulated the bivalves’ cellular immune response against *Vibrio.* Our results showed a significant positive effect of gill function on immunity in mussels fed *Vibrio* + *Alexandrium* (Fig. 2D), demonstrating the increased energetic demands of upregulating immune function. Several previous studies have also reported an increase in specific components of bivalves’ cellular immune response, such as significant increases in phagocytically active cells in clams exposed simultaneously to *Alexandrium* and *Vibrio*^46^,^47^. However, the exact mechanisms responsible currently remain unidentified. Our SEM analysis revealed that *Vibrio* exposure had complex and detrimental effects on metabolic and immune system function, which led to an increase in the toxin-pathogen load. Warming and hyposalinity of *Alexandrium*-fed mussels, on the other hand, clearly affected a specific immunometabolic pathway, which facilitated an overall increase in the toxin-pathogen load.

Despite the fact that our study was medium term (14 days) and was conducted in mesocosms we saw multiple statistically significant effects of simulated climate change and microorganism exposure on the physiology of *P. viridis*. This suggests that if this species is exposed to these combinations of conditions under future global change scenarios that there would be effects that would be detrimental to their fitness as well as to their overall toxicity. In tropical coastal regions, fish and shellfish are key sources of protein and income for local communities. In these areas even small-scale outbreaks of *Vibrio* and/or PST contamination can have devastating economic and social consequences. Clearly, further work is required to understand algal-bacterial-bivalve interactions for sustainable and economic management of economically and ecologically valuable species. As a first step, our results suggest that: *i*) the antagonistic *Vibrio-Alexandrium* relationship may be a valuable tool in the management of seafood safety, and; *ii)* toxic marine microbes can strongly modify the direct and indirect effects of climate change on commercially important shellfish. These findings provide a key stepping-stone toward increasing the resilience of coastal marine fisheries and aquaculture to the challenges posed by future climate change.

## Methods

### Experimental design and setup

To investigate the combined effects of warming and hyposalinity and exposure to toxic phytoplankton and pathogenic bacteria on various aspects of physiological function in *P. viridis* a nested orthogonal experimental design was used. This incorporated two levels of seawater temperature and two salinities within each microorganism exposure (Supplementary Fig. 2). The two seawater temperature levels (28 °C and 32 °C) correspond to the mean monthly sea surface temperature (SST) at the collection site^7^ and to an increase of +4 °C in line with projected warming trends for SST^1^. The two salinity levels (35 PSU and 31 PSU) correspond to the mean monthly sea surface salinity (SSS) at the collection site^7^ and to a decrease of 4 PSU as projected by future rainfall models^1^. The four microorganism exposures used were: the non-toxic diatom *Thalassiosira weissflogii* (1000 cells/mL), the PST producing dinoflagellate *Alexandrium minutum* (100 cells/mL), the pathogenic bacteria *Vibrio parahaemolyticus* (10,000 cells/mL) together with *T. weissflogii* (1000 cells/mL), and a combined treatment of *A. minutum* (100 cells/mL) and *V. parahaemolyticus* (10,000 cells/mL). *A. minutum* is a widely distributed species responsible for paralytic shellfish poisoning events in coastal regions around the world^48^, and has previously been recorded from the Mangalore coast^49^. *V. parahaemolyticus* is generally considered to be the most common cause of bacterial seafood contamination, several strains of which have been detected in India^50^,^51^,^52^,^53^. The concentration of *T. weissflogii* fed, 1000 cells/mL, corresponds to the amount used (based on carbon concentration) in similar experiments using mussels, a non-toxic algal feed and *A. minutum*^29^,^54^. The daily presence of faeces and pseudofaces was used as an indicator of sufficient feed concentration^55^. The amount of *A. minutum* used (100 cells/mL) is the number of cells needed to maintain the same concentration of carbon as 1000 cells/mL of *T. weissflogii*^56^,^57^. *Vibrio parahaemolyticus* (10,000 cells/mL) was fed together with *T. weissflogii* (1000 cells/mL) to avoid potential starvation of mussels fed solely with bacteria^58^.

Mussels were exposed for 14 days to one of four microorganism exposures detailed above nested in combinations of the four climate change treatments: ‘control’ (28 °C + 35 PSU), ‘warming’ (32 °C + 35 PSU), ‘hyposalinity’ (28 °C + 31 PSU), or ‘warming + hyposalinity’ (32 °C + 31 PSU). For the groups exposed to warming, the water temperature in the aquaria (described above) was raised by 1 °C per day until 32 °C was achieved (day zero for all experimental exposures), by using stick heaters. Similarly, for groups exposed to hyposalinity, salinity was decreased by 1 PSU per day until 31 PSU was obtained, by adjustment with freshwater that had been filtered by reverse osmosis beforehand. Mussels were randomly divided into four groups of 256, each assigned to one of the four exposure regimes described above. Each group was further sub-divided into four groups of 64 individuals and allocated to one of the four nested climate change treatments, and then each of these were subdivided again into four groups of 16 individuals each randomly allocated to one of four replicate aquaria, thus avoiding pseudoreplication. Aquaria (volume = 20 L) were filled with aerated seawater that had previously been sand filtered and ozonated. For these smaller aquaria, temperature was maintained above ambient by placing individual aquaria into larger tanks, the water of which was heated using stick heaters, thus avoiding any difficulties in temperature regulation in a small volume. Salinity was decreased below ambient by adjustment with freshwater as before. Stocking density was at a maximum of one mussel per litre. Mussels were fed daily as per their allocated microorganism exposure. All other experimental procedures were as previously described above.

At the end of the exposure period (after 14 d), the mussels from each sub-group, in each of the aquaria were subdivided into three groups (blinding not used). See supporting information for full details of animal husbandry, all experimental procedures and assay protocols. Individuals in group one were weighed. Haemolymph was then taken from the posterior abductor muscle and added to an equal volume of Hepes-buffered physiological saline for use in the Neutral Red Retention Assay to assess lysosomal membrane stability and thus immunobiological status. Mussels were then opened, sexed, and the mantle tissue was rapidly dissected and immediately snap frozen for determination of levels of ATP, ADP, AMP, glucose and glycogen and thus cellular energy status. Gill tissue was also collected, added to SEI buffer and snap frozen for determination of gill function via Na^+^/K^+^-ATPase activity. Mantle and gill tissue samples were stored at −80 °C until analysis. Group two mussels were used for determination of metabolic rates (MO_2_) and thus oxidative metabolism. Group three were used for toxin and/or bacterial concentration quantification to give a total combined toxin-pathogen load score (see Supplementary Materials for details of the calculation of the toxin-pathogen load score).

### General linear model (GLM) analyses

Mixed linear model tests were used to investigate the effect of microorganism exposure and climate change effects on the mean physiological traits investigated, with the term ‘tank’ as a random factor and individual body mass as covariate. If significant differences were found among different treatments, this was tested using the Tukey test. All data met assumptions for normality and for homogeneity of variances (Levene’s test, *P* > 0.05). In a preliminary analysis, the terms ‘tank’ and ‘body mass’ were shown not to have a significant effect on the parameters investigated, and were therefore removed. All remaining analyses were therefore completed using GLM. All analyses were conducted in SPSS v. 22. The power of our experiments (the likelihood that our tests would have detected a biologically meaningful effect had it existed) was determined by power analysis using the program G*Power^59^.

### Structural Equation Modelling

Since the presence of microorganisms resulted in four different scenarios (a control state with only non-toxic diatoms, exposure to *Vibrio*, exposure to *Alexandrium* and exposure to *Vibrio* + *Alexandrium*), data were separated into four groups – control group, *Vibrio, Alexandrium, Vibrio* + *Alexandrium* – and analysed with a multigroup Structural Equation Model (SEM)^60^. This allowed us to test whether climate change effects through the physiology and toxicity level of the mussels differed when exposed to different toxic and non-toxic microorganisms. In the multigroup SEM, we assessed the effects of three binary predictor variables – (i) warming (0/1), (ii) hyposalinity (0/1) and (iii) warming + hyposalinity (0/1) – and modelled their direct and/or indirect effects on six continuous response variables: (i) gill function, (ii) glycogen concentration, (iii) oxygen consumption, (iv) ATP concentration, (v) immunity (as lysosomal membrane stability measured as neutral red retention) and (vi) toxicity. Direct effects are when variable A will cause variable B to change (e.g. warming → O_2_ consumption), whereas indirect effects are the sum of all possible variables affecting variable B (e.g., the indirect effect of warming on O_2_ consumption is the product of the path between warming → gill function, gill function → O_2_ consumption, warming → glycogen, glycogen → O_2_ consumption) (see also Fig. 2 and Supplementary Tables 3 and 4).

First, we analysed the data in each group to ensure that the basic structure of the model was consistent with the data^61^. Data were analysed by comparing models with the observed covariance matrix, using maximum likelihood and Chi-square as goodness of fit measures. Data were considered significantly different from the model when *P* < 0.05. Since data from the individual groups fit the model (*P > *0.05), we deemed it legitimate to perform a multigroup SEM analysis. All variables in the model were initially constrained to vary equally across all groups. Standardised residual covariances, which display the difference between sample covariance and implied covariance, were then examined to locate variable inequalities between groups. Any inequalities that differed between the four groups by >2 in absolute values were relaxed (or allowed to vary freely across groups), and the analysis was run again. This stepwise procedure was performed until the model Chi-square no longer changed^60^,^61^. Intercepts of the regression equations were also investigated to test differences for each endogenous variable between the four groups (Supplementary Table 3). Significance levels for individual paths between variables were set at *α* = 0.05. Structural equation models were run in AMOS (v. 20).

## Additional Information

**How to cite this article**: Turner, L. M. *et al*. Pathogenic marine microbes influence the effects of climate change on a commercially important tropical bivalve. *Sci. Rep.* **6**, 32413; doi: 10.1038/srep32413 (2016).

## Supplementary Material

Supplementary Information

## Figures and Tables

**Table 1:** GLM results of the effects of simulated climate change and microorganism exposure and their interaction on mussel toxicity, immunobiological status, oxidative metabolism, gill function and cellular energy status in the green mussel *Perna viridis.*

Parameter	Climate change	Microorganism exposure	Climate change + Microorganism exposure
*Toxicity*			
Toxin-pathogen load score	*F*_*3*_ = 1.057*, P* = 0.369	*F*_*3*_ = 1.760*, P* = 0.157	***F***_***9***_ = **1.953*****, P*** = **0.048**
PST	***F***_**3**_ = **5.155,** ***P*** = **0.003**	***F***_**2**_ = **4.650,** ***P*** = **0.013**	***F***_**6**_ = **5.573,** ***P*** = **0.000**
*Vibrio* (RT-PCR)	*F*_3_ = 0.299, *P* = 0.826	*F*_2_ = 0.332, *P* = 0.718	*F*_5_ = 0.412, *P* = 0.840
*Immunobiological status*			
Neutral red retention	***F***_**3**_ = **14.344,** ***P*** = **0.000**	***F***_**3**_ = **13.593,** ***P*** = **0.000**	***F***_**9**_ = **3.476,** ***P*** = **0.001**
*Oxidative metabolism*			
O_2_ uptake *Gill function*	*F*_3_ = 1.860, *P* = 0.138	***F***_**3**_ = **10.422,** ***P*** = **0.000**	***F***_**9**_ = **2.694,** ***P*** = **0.006**
Na^+^/K^+^-ATPase	*F*_3_ = 1.223, *P* = 0.303	*F*_3_ = 1.169, *P* = 0.323	***F***_**9**_ = **1.985,** ***P*** = **0.044**
*Cellular energy status*			
ATP	*F*_3_ = 1.096, *P* = 0.352	***F***_**3**_ = **31.410,** ***P*** = **0.000**	***F***_**9**_ = **9.886,** ***P*** = **0.000**
ADP	*F*_3_ = 1.789, *P* = 0.151	***F***_**3**_ = **17.486,** ***P*** = **0.000**	***F***_**9**_ = **5.860,** ***P*** = **0.000**
AMP	***F***_**3**_ = **3.181,** ***P*** = **0.025**	***F***_**3**_ = **76.915,** ***P*** = **0.000**	***F***_**9**_ = **4.518,** ***P*** = **0.000**
AEC	***F***_**3**_ = **2.757,** ***P*** = **0.044**	***F***_**3**_ = **60.820,** ***P*** = **0.000**	***F***_**9**_ = **7.311,** ***P*** = **0.000**
TAN	*F*_3_ = 2.091, *P* = 0.103	***F***_**3**_ = **35.782,** ***P*** = **0.000**	***F***_**9**_ = **4.024,** ***P*** = **0.000**
Glucose	*F*_3_ = 2.544, *P* = 0.057	***F***_**3**_ = **99.367,** ***P*** = **0.000**	*F*_9_ = 1.314, *P* = 0.232
Glycogen	*F*_3_ = 2.404, *P* = 0.069	***F***_**3**_ = **13.537,** ***P*** = **0.000**	*F*_9_ = 1.753, *P* = 0.080

Detailed test results for statistically significant effects of simulated climate change, and/or microorganism exposure are shown in Tables 2 and 3. *F*-values are given with degrees of freedom in subscript. Significant values (*P* < 0.05) are highlighted in bold. AEC, adenylate energy change. TAN, total adenylate nucleotides.

**Table 2:** Parameter estimates and Tukey post-hoc test results of the effects of simulated climate change (warming, hyposalinity and warming + hyposalinity) on mussel toxicity, immunobiological status and cellular energy status in the green mussel *Perna viridis.*

Parameter	Warming	Hyposalinity	Warming + Hyposalinity
*Toxicity*			
PST (μg STX eq kg^−1^)	**0.348,** ***T*** = **3.36,** ***P*** = **0.007**	**0.138,** ***T*** = **3.02,** ***P*** = **0.019**	**0.232,** ***T*** = **2.86,** ***P*** = **0.029**
*Immunobiological status*			
Neutral red retention (min)	**54.58,** ***T*** = **4.845,** ***P*** = **0.001**	**68.84,** ***T*** = **3.598,** ***P*** = **0.024**	**31.61,** ***T*** = **6.570,** ***P*** = **0.000**
*Cellular energy status*			
AMP (μmol g^−1^)	0.872, *T* = 1.44, *P* = 0.474	**0.752,** ***T*** = **3.01,** ***P*** = **0.016**	0.923, *T* = 0.48, *P* = 0.963
AEC	0.564, *T* = 2.21, *P* = 0.123	**0.574,** ***T*** = **2.59,** ***P*** = **0.050**	0.552*, T* = 1.12, *P* = 0.678

Results shown only for Parameters with statistically significant effects (Table 1).

*T*-values are given. Significant values (*P* < 0.05) are highlighted in bold. AEC, adenylate energy change.

**Table 3:** Parameter estimates and Tukey post-hoc test results of the effects of microorganism exposure (*Alexandrium, Vibrio* and *Alexandrium* + *Vibrio*) on mussel toxicity, immunobiological status, oxidative metabolism and cellular energy status in the green mussel *Perna viridis.*

Parameter	Alexandrium	Vibrio	Alexandrium + Vibrio
Toxicity			
PST (μg STX eq kg^−1^)	**3.587, T** = **2.88, P** = **0.015**	NA	0.269, T = 0.09, P = 0.996
Immunobiological status			
Neutral red retention (min)	**47.076, T** = **5.148, P** = **0.001**	**39.241, T** = **6.112, P** = **0.000**	**68.772, T** = **3.691, P** = **0.038**
Oxidative metabolism			
O_2_ uptake (μmol O_2_ h^−1^ g^−1^)	**6.923, T** = **2.114, P** = **0.012**	**10.419, T** = **5.564, P** = **0.000**	6. 602, T = 2.082, P = 0.134
Cellular energy status			
ATP (μmol g^−1^)	0.866, T = 2.46, P = 0.069	**1.372, T** = **7.38, P** = **0.000**	**1.249, T** = **4.38, P** = **0.000**
ADP(μmol g^−1^)	1.002, T = 1.47, P = 0.458	**0.757, T** = **6.73, P** = **0.000**	1.05, T = 0.90, P = 0.806
AMP(μmol g^−1^)	**0.907, T** = **3.47, P** = **0.004**	0.519, T = 2.01, P = 0.186	**1.44, T** = **12.29, P** = **0.000**
AEC	**0.507, T** = **3.63, P** = **0.002**	**0.662, T** = **7.25, P** = **0.000**	**0.488, T** = **5.65, P** = **0.000**
TAN	2.775, T = 0.13, P = 0.999	2.648, T = 1.05, P = 0.723	**3.739, T** = **8.50, P** = **0.000**
Glucose (μmol g^−1^)	**1.113, T** = **12.01, P** = **0.000**	**2.855, T** = **13.17, P** = **0.000**	**0.721, T** = **14.78, P** = **0.000**
Glycogen (μmol g^−1^)	**98.006, T** = **3.04, P** = **0.014**	**59.093, T** = **6.27, P** = **0.000**	123.374, T = 2.25, P = 0.113

Results shown only for Parameters with statistically significant effects (Table 1).

*T*-values are given. Significant values (*P* < 0.05) are highlighted in bold. AEC, adenylate energy change. TAN, total adenylate nucleotides.

**Table 4:** SEM statistics.

Models	*χ*^*2*^	df	*P*
Individual group models			
Control group, diatoms	0.08	1	0.77
*Vibro* treatment	0.02	1	0.89
*Alexandrium* treatment	2.82	1	0.10
Vibro + *Alexandrium* treatment	0.83	1	0.36
Multigroup model	18.9	20	0.53

Chi-square (*χ*^2^) likelihood tests for the individual and multigroup SEM models. When *P* ≥ 0.05, fitted models are not significantly different from observed data.
