# Supplementary material for: Pathogenic marine microbes influence the effects of climate change on a commercially important tropical bivalve
Source: Sci Rep. 2016 Aug 31;6:32413. doi: 10.1038/srep32413 (PMC5006160; doi:10.1038/srep32413)
Supplement: Supplementary Information [file srep32413-s1.pdf]

Supplementary materials for ‘Pathogenic marine microbes influence the effects of climate change on a commercially important tropical bivalve’

Lucy M. Turner, Christian Alsterberg, Andrew D. Turner, Girisha S.K., Ashwin Rai, Jonathan N. Havenhand, M.N. Venugopal, Indrani Karunasagar, Anna Godhe

Supplementary Figure

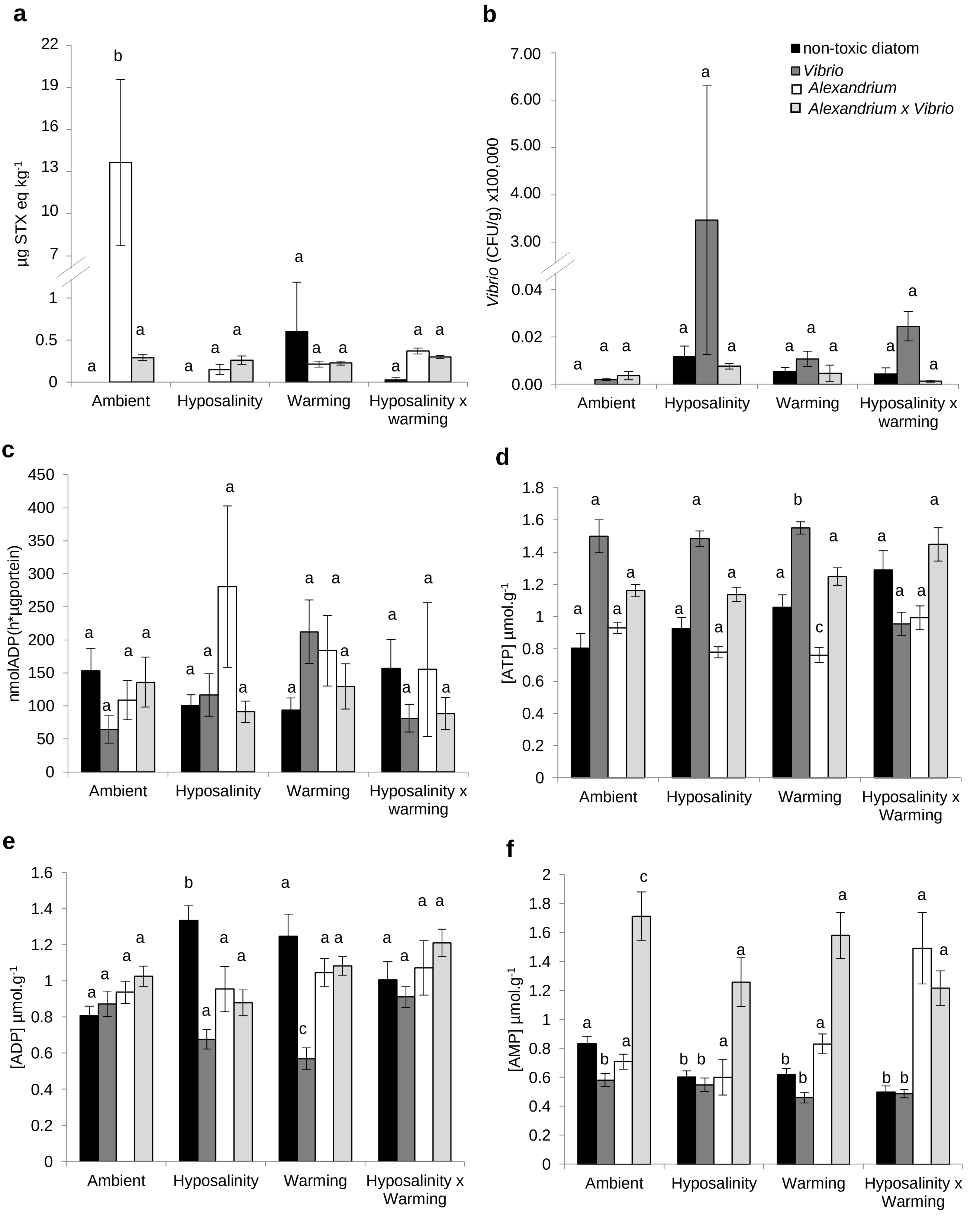

Supplementary Figure 1 | The interactive effects of microorganism exposure and simulated climate change effects on some aspects of physiological function in *P. viridis*. The (**a**) [PST], (**b**) bacterial load, (***c***) gill function, (**d**) mantle [ATP] (µmol g^{-1}), (***e***) mantle [ADP] (µmol g^{-1}), and (**f)** mantle [AMP] (µmol g^{-1}) of *Perna viridis* following 14 day's exposure to the non-toxin producing diatom *Thalassiosira weissflogii,* the toxin producing dinoflagellate *Alexandrium minutum* and/or the pathogenic bacteria *Vibrio parahaemolyticus* (fed together with *T. weissflogii*) under differing climate change conditions. Within each graph, different letters indicate means that are significantly different from each other (P < 0.05), but see also Tables 1-3. Data are means ± SEM. Samples sizes (n=16) but see Supplementary Table 2.

Supplementary Tables

Supplementary Table 1 | Survival rates for *Perna viridis* after 14 d exposure to different simulated climate change conditions and microorganisms.

Microorganism exposure	Climate change	% Survival
Non-toxic diatom	Ambient	100
	Hyposalinity	100
	Warming	95.21
	Hyposalinity + warming	70.31
Alexandrium	Ambient	76.56
	Hyposalinity	45.31
	Warming	70.31
	Hyposalinity + warming	17.19
Vibrio	Ambient	100
	Hyposalinity	100
	Warming	89.06
	Hyposalinity + warming	93.75
Alexandrium + *Vibrio*	Ambient	90.63
	Hyposalinity	87.50
	Warming	48.44
	Hyposalinity + warming	92.19

Supplementary Table 2 | Sample sizes (n) for each analysis conducted on *Perna viridis* after 14 d exposure to different simulated climate change conditions and microorganisms.

		Toxicity			Immunobiological status	Oxidative metabolism	Gill function	Cellular energy status
Microorganism exposure	Climate change	Toxin-pathogen load	PST	Vibrio	Neutral red retention	O_2 uptake	Na^+/K^+-ATPase	ATP, ADP, AMP, AEC, TAN, Glucose, Glycogen
Non-toxic diatom	Ambient	8	8	8	16	16	14	16
	Hyposalinity	16	8	8	16	16	15	16
	Warming	16	8	8	16	14	13	16
	Hyposalinity + warming	12	8	4	12	12	12	12
Alexandrium	Ambient	6	6	NA	16	10	13	16
	Hyposalinity	4	4	NA	4	4	3	4
	Warming	14	11	NA	14	15	13	14
	Hyposalinity + warming	3	3	NA	4	4	2	4
Vibrio	Ambient	15	NA	15	16	16	11	16
	Hyposalinity	16	NA	16	16	16	12	16
	Warming	16	NA	16	15	16	13	15
	Hyposalinity + warming	16	NA	16	16	16	11	16
Alexandrium + Vibrio	Ambient	12	6	6	15	16	15	15
	Hyposalinity	11	3	8	13	16	13	13
	Warming	9	5	4	12	13	10	12
	Hyposalinity + warming	6	3	3	7	12	3	7

Where sample size n<16 this was the result of mussel mortality during 14 day exposure to different simulated climate change conditions and microorganisms (Supplementary Table 1). For toxicity determination, samples were pooled (see supplementary methods for details.)

Supplementary Table 3 | Investigation of model intercepts between groups with chi squares ($\chi 2$).

Variable	Intercept different across groups	Intercept equal across groups	Difference in model $\chi 2$
Gill function	18.9	21.6	2.7
Glycogen	18.9	33.6	14.7
O_2 consumption	18.9	21.6	2.7
ATP	18.9	50	31.1
NRRT	18.9	33.6	14.7
Toxin-pathogen load	18.9	24.9	6

First, all intercepts were allowed to differ across groups. Second, each variable was investigated by setting the intercept as equal across groups, which changed the model $\chi 2$. The difference in model $\chi 2$ indicates whether the intercept is significantly different across groups.

Supplementary Table 4a | Standardised total, direct and indirect effects for the control group.

	Salinity	Temp	T×S	Glyco.	Gill f.	O_2 c.	ATP	NRRT
Standardized total effects								
Glycogen	-0.051	-0.402	0.270	0.000	0.000	0.000	0.000	0.000
Gill function	0.194	0.063	0.232	0.257	0.000	0.000	0.000	0.000
O_2 consumption	-0.180	-0.592	0.245	0.043	-0.003	0.000	0.000	0.000
ATP	0.191	0.241	0.208	0.000	0.000	-0.134	0.000	0.000
NRRT	-0.120	-0.866	-0.028	-0.030	0.001	0.004	0.091	0.000
Toxin-pathogen load	0.042	0.360	-0.267	-0.354	-0.105	-0.081	0.086	-0.168
Standardized direct effects								
Glycogen	-0.051	-0.402	0.270	0.000	0.000	0.000	0.000	0.000
Gill function	0.207	0.166	0.162	0.257	0.000	0.000	0.000	0.000
O_2 consumption	-0.177	-0.574	0.234	0.044	-0.003	0.000	0.000	0.000
ATP	0.167	0.164	0.239	0.006	0.000	-0.134	0.000	0.000
NRRT	-0.136	-0.891	-0.043	-0.031	0.001	0.016	0.091	0.000
Toxin-pathogen load	-0.006	0.024	-0.163	-0.329	-0.105	-0.067	0.101	-0.168
Standardized indirect effects								
Glycogen	0.000	0.000	0.000	0.000	0.000	0.000	0.000	0.000
Gill function	-0.013	-0.104	0.069	0.000	0.000	0.000	0.000	0.000
O_2 consumption	-0.003	-0.018	0.011	-0.001	0.000	0.000	0.000	0.000
ATP	0.024	0.077	-0.031	-0.006	0.000	0.000	0.000	0.000
NRRT	0.016	0.025	0.015	0.001	0.000	-0.012	0.000	0.000
Toxin-pathogen load	0.048	0.336	-0.104	-0.025	0.000	-0.014	-0.015	0.000

Total effects are the sum of all direct and indirect effects. Direct effects are the direct effects of one variable on another variable, and indirect effects are sum of all products affecting one variable (e.g., the indirect effect of Temp on O_2 consumption is the product of the path between Temp → Gill function, Gill function → O_2 consumption, Temp → Glycogen, Glycogen → O_2 consumption). Temp (temperature), T×S (Temperature × Salinity), Glyco. (glycogen), Gill f. (gill function), O_2 c. (oxygen consumption), NRRT (immunity).

Supplementary Table 4b | Standardised total, direct and indirect effects for the *Vibrio* group.

	Salinity	Temp	T×S	Glyco.	Gill f.	O_2 c.	ATP	NRRT
Standardized total effects								
Glycogen	-0.069	-0.455	0.361	0.000	0.000	0.000	0.000	0.000
Gill function	0.125	0.249	-0.359	-0.138	0.000	0.000	0.000	0.000
O_2 consumption	0.424	-0.033	0.121	0.163	0.390	0.000	0.000	0.000
ATP	-0.362	-0.178	-0.467	0.023	-0.011	-0.028	0.000	0.000
NRRT	-0.704	-0.600	0.488	0.133	0.116	-0.083	0.271	0.000
Toxin-pathogen load	0.163	-0.080	-0.209	0.021	0.339	-0.437	-0.337	-0.308
Standardized direct effects								
Glycogen	-0.069	-0.455	0.361	0.000	0.000	0.000	0.000	0.000
Gill function	0.115	0.186	-0.309	-0.138	0.000	0.000	0.000	0.000
O_2 consumption	0.391	-0.031	0.183	0.216	0.390	0.000	0.000	0.000
ATP	-0.348	-0.167	-0.474	0.028	0.000	-0.028	0.000	0.000
NRRT	-0.582	-0.518	0.619	0.159	0.148	-0.075	0.271	0.000
Toxin-pathogen load	0.000	-0.363	0.000	0.221	0.555	-0.470	-0.253	-0.308
Standardized indirect effects								
Glycogen	0.000	0.000	0.000	0.000	0.000	0.000	0.000	0.000
Gill function	0.009	0.063	-0.050	0.000	0.000	0.000	0.000	0.000
O_2 consumption	0.034	-0.002	-0.062	-0.054	0.000	0.000	0.000	0.000
ATP	-0.014	-0.012	0.007	-0.005	-0.011	0.000	0.000	0.000
NRRT	-0.122	-0.082	-0.131	-0.026	-0.032	-0.008	0.000	0.000
Toxin-pathogen load	0.163	0.283	-0.209	-0.200	-0.216	0.033	-0.084	0.000

Total effects are the sum of all direct and indirect effects. Direct effects are the direct effects of one variable on another variable, and indirect effects are sum of all products affecting one variable (e.g., the indirect effect of Temp on O_2 consumption is the product of the path between Temp → Gill function, Gill function → O_2 consumption, Temp → Glycogen, Glycogen → O_2 consumption). Temp (temperature), T×S (Temperature × Salinity), Glyco. (glycogen), Gill f. (gill function), O_2 c. (oxygen consumption), NRRT (immunity).

Supplementary Table 4c | Standardised total, direct and indirect effects for the *Alexandrium* group.

	Salinity	Temp	T×S	Glyco.	Gill f.	O_2 c.	ATP	NRRT
Standardized total effects								
Glycogen	-0.072	-0.209	0.346	0.000	0.000	0.000	0.000	0.000
Gill function	0.455	0.080	-0.344	0.055	0.000	0.000	0.000	0.000
O_2 consumption	-0.275	0.087	-0.250	0.582	0.038	0.000	0.000	0.000
ATP	-0.287	-0.434	0.762	-0.120	-0.020	-0.522	0.000	0.000
NRRT	-0.246	-0.524	0.173	-0.104	0.249	-0.451	0.461	0.000
Toxin-pathogen load	-0.301	-0.304	0.175	0.039	0.120	-0.344	0.397	0.948
Standardized direct effects								
Glycogen	-0.072	-0.209	0.346	0.000	0.000	0.000	0.000	0.000
Gill function	0.459	0.091	-0.362	0.055	0.000	0.000	0.000	0.000
O_2 consumption	-0.251	0.205	-0.438	0.580	0.038	0.000	0.000	0.000
ATP	-0.418	-0.350	0.568	0.184	0.000	-0.522	0.000	0.000
NRRT	-0.288	-0.315	-0.160	0.059	0.266	-0.210	0.461	0.000
Toxin-pathogen load	-0.001	0.201	-0.019	0.103	-0.119	0.063	-0.040	0.948
Standardized indirect effects								
Glycogen	0.000	0.000	0.000	0.000	0.000	0.000	0.000	0.000
Gill function	-0.004	-0.011	0.019	0.000	0.000	0.000	0.000	0.000
O_2 consumption	-0.024	-0.118	0.187	0.002	0.000	0.000	0.000	0.000
ATP	0.130	-0.084	0.194	-0.303	-0.020	0.000	0.000	0.000
NRRT	0.042	-0.209	0.333	-0.163	-0.017	-0.240	0.000	0.000
Toxin-pathogen load	-0.300	-0.505	0.194	-0.064	0.239	-0.406	0.437	0.000

Total effects are the sum of all direct and indirect effects. Direct effects are the direct effects of one variable on another variable, and indirect effects are sum of all products affecting one variable (e.g., the indirect effect of Temp on O_2 consumption is the product of the path between Temp → Gill function, Gill function → O_2 consumption, Temp → Glycogen, Glycogen → O_2 consumption). Temp (temperature), T×S (Temperature × Salinity), Glyco. (glycogen), Gill f. (gill function), O_2 c. (oxygen consumption), NRRT (immunity).

Supplementary Table 4d | Standardised total, direct and indirect effects for the *Vibrio* × *Alexandrium* group.

	Salinity	Temp	T×S	Glyco.	Gill f.	O_2 c.	ATP	NRRT
Standardized total effects								
Glycogen	-0.075	-0.096	0.384	0.000	0.000	0.000	0.000	0.000
Gill function	0.035	0.166	-0.300	0.093	0.000	0.000	0.000	0.000
O_2 consumption	0.258	-0.086	-0.438	-0.009	-0.025	0.000	0.000	0.000
ATP	-0.005	0.139	0.512	-0.193	0.000	-0.002	0.000	0.000
NRRT	-0.524	-0.091	0.047	0.232	0.159	0.070	-0.295	0.000
Toxin-pathogen load	0.082	-0.035	-0.282	-0.138	-0.080	-0.082	0.225	-0.216
Standardized direct effects								
Glycogen	-0.075	-0.096	0.384	0.000	0.000	0.000	0.000	0.000
Gill function	0.042	0.175	-0.336	0.093	0.000	0.000	0.000	0.000
O_2 consumption	0.258	-0.083	-0.443	-0.006	-0.025	0.000	0.000	0.000
ATP	-0.019	0.120	0.585	-0.193	0.000	-0.002	0.000	0.000
NRRT	-0.537	-0.056	0.215	0.160	0.161	0.069	-0.295	0.000
Toxin-pathogen load	-0.015	-0.080	-0.377	-0.053	-0.048	-0.067	0.161	-0.216
Standardized indirect effects								
Glycogen	0.000	0.000	0.000	0.000	0.000	0.000	0.000	0.000
Gill function	-0.007	-0.009	0.036	0.000	0.000	0.000	0.000	0.000
O_2 consumption	0.000	-0.003	0.005	-0.002	0.000	0.000	0.000	0.000
ATP	0.014	0.019	-0.073	0.000	0.000	0.000	0.000	0.000
NRRT	0.013	-0.036	-0.168	0.071	-0.002	0.000	0.000	0.000
Toxin-pathogen load	0.098	0.045	0.095	-0.085	-0.033	-0.015	0.064	0.000

Total effects are the sum of all direct and indirect effects. Direct effects are the direct effects of one variable on another variable, and indirect effects are sum of all products affecting one variable (e.g., the indirect effect of Temp on O_2 consumption is the product of the path between Temp → Gill function, Gill function → O_2 consumption, Temp → Glycogen, Glycogen → O_2 consumption). Temp (temperature), T×S (Temperature × Salinity), Glyco. (glycogen), Gill f. (gill function), O_2 c. (oxygen consumption), NRRT (immunity).

Additional details for Methods

Calculation of toxin-pathogen load score

Paralytic shellfish toxin (PST) content of mussels was measured in µgSTX di-HCl eq/kg shellfish tissue and *Vibrio* concentration in CFU/g. For each mussel sample, one, or both of these values were normalised (depending on exact microorganism exposure regime) and added together where appropriate, to give an overall value per individual for toxin-pathogen load (see Supplementary Tables 5 and 6).

Supplementary Table 5 | Mussel paralytic shellfish toxin (PST) concentration normalisation.

Total paralytic shellfish toxin (PST) (µgSTX di- HCl eq/kg)	Normalisation score
0	0
0.01-1	1
1.01-2	2
2.01-3	3
3.01-4	4
4.01-5	5
5.01-6 etc.	6 etc.

Supplementary Table 6 | Mussel *Vibrio* concentration normalisation.

Vibrio (CFU/g)	Normalisation score
0	0
0.01-1000	1
1001-2000	2
2001-3000	3
3001-4000	4
4001-5000	5
5001-6000 etc.	6 etc.

Animal collection and husbandry

Mussels (*Perna viridis,* Linnaeus, 1758) (27.41 ± 0.49 g wet mass) were collected from Someshwara, Mangalore, India (12° 47’ 15.64” N, 74° 51’ 8.67” E). Individuals were immediately transferred to the experimental aquarium facility <60 min after collection. Upon arrival mussels were exposed to constant conditions for at least five

days to remove any effects of differences in recent environmental history. This was achieved by placing the mussels in a number of aquaria (volume= 200 L) filled with aerated seawater *T* (°C) = 28, *S* (PSU) = 35 that had previously been sand filtered and ozonated to remove the microbial community. Stocking density was at a maximum of one mussel per two litres. Mussels were exposed to a 12h:12h L:D cycle and fed the non-toxin producing diatom *Thalassiosira weissflogii* (Instant Algae TW1200, Reed Mariculture Inc, Campbell, CA, USA) once daily at a concentration of 1000 cells/mL. Every 1-2 d faeces and pseudofaeces were removed from the aquaria and half of the water was exchanged to remove metabolic waste products. Dead mussels were removed daily, however in most cases overall mortality was negligible (see Supplementary Table 1 for details). Temperature and salinity were measured daily and corrected if needed.

Culture of microorganisms

A strain of *A. minutum* (CCMP113) was obtained from the Gothenburg University Culture Collection, Gothenburg, Sweden. The algae were cultured on site in f/2 medium[1] based on 0.5 µm filtered natural seawater adjusted to 26 PSU at 25 °C on a 12h:12h L:D cycle and irradiance of 50 µmol photons m^{-2} s^{-1}.

A pathogenic strain of *Vibrio parahaemolyticus* (VP7)[2] was procured from the culture collections maintained at the Department of Fisheries Microbiology, College of Fisheries, Mangalore. The strain was retrieved from frozen (-80 °C) in tryptone soy broth containing 1% NaCl (TSBS) by incubating overnight at 30 °C. The young *V. parahaemolyticus* culture was then sub-cultured into fresh TSBS medium and the optical density (OD_{600}) of which was measured every 30 min spectrophotometrically. Simultaneously, aliquots were drawn for viable count determination by plating on tryptone soy agar with 1% NaCl (TSAS). The plates were incubated overnight at 30 °C. A growth curve was prepared by plotting the viable count (x-axis) against OD_{600} values (y-axis). This graph was then used to determine the viable cell count.

Lysosome membrane stability

Lysosome membrane stability was evaluated in mussel haemocytes using the Neutral Red Retention Assay[3,4]. In healthy mussels lysosomes will retain the acidophilic vital dye neutral red. Cellular toxicity will result in the integrity of the membrane being compromised and subsequent leakage of the dye[5]. Briefly glass

microscope slides were coated with 5 % poly-L-lysine and left to dry in a humid chamber for 30 min. Next, for each sample, 40 µL of the haemolymph-saline mixture (as previously desribed) was added in the same position where the poly-L-lysine was added. Slides were placed into a dark humid chamber for 30 min to allow cells to attach. After 30 min the slides were placed onto their side to allow excess solution to run off. Next 40 µL of a freshly prepared neutral red working solution (5 µL/mL saline from a stock solution of 20 mg neutral red dye dissolved in 1 mL DMSO) was added to the area containing the attached cells and a cover slip was applied. Slides were incubated for 15 min and then inspected under a microscope (40X) at increasing intervals (15-60 min) for a total of 180 min to determine at what point in time the dye that had been readily taken up into the lysosomal compartment of the cells, was lost to the remainder of the cytosol. The test for an individual sample was terminated when the lysosomal dye loss was evident in 50% (numerically assessed per field of view) of the haemocytes, and the time recorded. Data are expressed as Neutral Red Retention Time[3].

Determination of gill Na^+/K^+-ATPase activity

Gill samples were defrosted on ice, pulse centrifuged and the SEI buffer decanted, following which samples were transferred to 2 mL microcentrifuge tubes. Next, 1.2 mL SEI deoxycholate buffer was added (0.1 % Na deoxycholic acid in SEI buffer) and the samples were homogenised with a bead beater (MM300, Retsch, Düsseldorf, Germany) using glass beads (Sigma-Aldrich, Poole, UK). The supernatant was analysed for Na^+/K^+-ATPase activity[6] in a microplate format using a Varioskan Flash plate reader (Thermo Scientific, Waltham, MA USA) equipped with proprietary software (SkanIt Software 2.4.3).

Determination of metabolite concentration

Mantle levels of ATP, ADP, AMP, glucose and glycogen were determined using standard assays. Beforehand mantle extracts were prepared[7,8]. Briefly, mantle samples were removed from storage at -80 °C and a known mass was transferred to chilled 2 mL microcentrifuge tubes. Next, the samples were homogenised with four parts 0.9 mol L^{-1} $HClO_4$ using a bead beater and glass beads, following which the homogenate was centrifuged for 10 min at 20,000 *g* at 4 ºC. The homogenate was then transferred to a second 2 mL microcentrifuge tube. To neutralise the effects of the acid, four parts K_2CO_3, 3.75 mol L^{-1} for five parts of $HClO_4$ was added and the

tubes were then centrifuged for 10 min at 20,000 *g* at 4 ºC. The resulting supernatant was removed and used for the following assays.

Concentrations of ATP, ADP, AMP, glucose and glycogen were determined spectrophotometrically in the mantle samples. All assays were undertaken in a microplate format using a Varioskan Flash plate reader. Mantle ATP, ADP and AMP concentrations were determined using NADH linked assays[8]. Glucose and glycogen concentrations were measured using the hexokinase method[7]. Glycogen was measured as glucose (hexokinase method) before and after treatment with glycoamylase. The total adenylate content (TAN) for each individual was calculated by summing the concentrations of the three adenylates measured (ATP, ADP and AMP). The adenylate energy charge (AEC) was calculated using the equation: AEC = (ATP+½ADP)/(ATP+ADP+AMP).

Determination of standard MO_2

After the 14 d feeding and abiotic exposure period, mussels were placed in aerated, clean (sand filtered, ozonated and GF/F filtered (0.45 µm)) seawater in individual 1 L aquaria containing *T. weissflogii* (1000 cells/mL) for 12 h to allow for defecation of unassimilated *A. minutum, V. parahaemolyticus* and/or *T. weissflogii* from the guts.

After this period, standard MO_2 was determined using closed respirometry. Respirometry chambers (volume = 500 mL) were filled with aerated, clean (sand filtered, ozonated and GF/F filtered) seawater at the respective experimental temperature and salinity and a magnetic flea added. A platform (50 x 50 x 15 mm) above the magnetic flea prevented contact between the mussel and the magnetic flea. A mussel was added to each chamber, which was then sealed while submerged to prevent air bubbles. The chambers were loosely covered with aluminium foil to ensure that disturbance to the mussel was minimised. Chambers were then placed onto magnetic stirrers (Remi Laboratory Instruments, Mumbai, India) to ensure adequate mixing of seawater and to prevent stratification of oxygen within the chamber. Before measurements began mussels were allowed to settle in the chambers for 1 h, which is the minimum time required for establishing resting MO_2. Planar optode spots (diameter 0.5 cm; PreSens Precision Sensing GmbH, Regensburg, Germany) were glued to the inside of each chamber. Oxygen levels in the chambers were measured every 5 min for a period of 1 h using a Fibox 4 oxygen

meter (PreSens Precision Sensing GmbH) and PreSens Datamanager software (PreSens Precision Sensing GmbH). All the equipment was located inside rooms where the appropriate temperature level was maintained. For each chamber the decline in pO_2 was linear over the measurement period and was never allowed to fall to hypoxic levels. Background respiration was taken into account by running blanks, and the average value across a number of blanks was subtracted from the original MO_2 value. MO_2 was expressed as µmol O_2 $h^{-1} \cdot g^{-1}$. Upon completion of measurements of MO_2, mussels were removed from the chambers, gently blotted dry and weighed. Mussel volume was also obtained by displacement.

Toxin extraction and analysis

Analysis of PST was conducted using liquid chromatography with tandem mass spectrometry (LC-MS/MS). Instrument solvents used for mobile phase preparation were of LC-MS-grade (Fisher Optima, ThermoFisher, UK) and all chemicals were LC-MS reagent grade where possible. All other reagents were HPLC grade. Mussels were first shucked and homogenised. Tissue homogenates were subjected to a single dispersive extraction using 1% acetic acid[9], with post-centrifuge supernatants desalted using pre-conditioned Supelclean ENVI-Carb 250mg/3 mL solid phase extraction (SPE) cartridges. 100 µL of SPE eluant from each sample was diluted in 300 µL acetonitrile prior to LC-MS/MS analysis. Chromatographic separation of PST was performed using Hydrophilic Interaction Liquid Chromatography (HILIC). A Waters Acquity UPLC I-Class was used with a 1.7 µm, 2.1x150 mm Waters Acquity BEH Amide UPLC column in conjunction with a Waters VanGuard BEH Amide guard cartridge. The columns were held at +60°C, with samples held in the autosampler at +4°C. MS/MS detection was conducted using a Waters Xevo TQ-S tandem quadrupole mass spectrometer. Selected reaction monitoring (SRM) was conducted to enable the detection and quantitation of 19 different saxitoxins analogues[10]. Quantitation of toxins in mussel tissue extracts was performed through external calibration using toxin standard calibrants prepared at six different concentration levels from certified reference toxin solutions (Institute of Biotoxin Metrology, National Research Council Canada (NRCC), Halifax, Nova Scotia, Canada). Toxin concentrations were calculated in µmoles/kg shellfish tissue and converted to saxitoxin dihydrochloride equivalents per kg shellfish tissue (STX di-HCl eq / kg) using toxicity equivalence factors (TEFs) taken from EFSA recommendations[11].

Vibrio uptake quantification by qPCR

Quantification of *V. parahaemolyticus* uptake by mussels was determined using qPCR. For use in qPCR, initially genomic DNA was extracted from 1 g of homogenised mussel soft tissue using alkaline lysis and treatment with cetyltrimethylammonium bromide–NaCl and phenol-chloroform, followed by ethanol precipitation[12]. After precipitation, the DNA pellet was washed with 70% ethanol, dried and resuspended in Tris-EDTA (1x TE) buffer. The concentration and purity of extracted DNA was examined using a NanoDrop® spectrophotometer (ND-1000, V3.3.0, Thermo Fisher Scientific, USA). DNA was then stored at -20 °C until use. Absolute quantification of *V. parahaemolyticus* was estimated using SYBR Green chemistry on a StepOnePlus™ qPCR system (Applied Biosystems, USA) by targeting the *tdh* gene[13]. Recombinant *tdh* plasmid (laboratory stock, Department of Fisheries Microbiology, College of Fisheries, Mangalore) with known copy number (4.38 x 10^{11}) was used as a standard[14]. The qPCR amplification was performed in 96-well plates (MicroAmp™; Applied Biosystems, USA) with a total volume of 25 µl in triplicates, containing 12.5 µl SYBR® Green PCR Mastermix, 2.5 pmol of each primer (D3 5`-CCACTACCACTCTCATATGC- 3`and D5 5`-GGTACTAAATGGCTGACATC-3`) and 80ng DNA template. The amplification conditions comprised an initial activation step at 50 °C for 2 min, initial denaturation at 95 °C for 10 min followed by 45 cycles of denaturation at 95 °C for 20 s, primer annealing at 55 °C for 20 s and elongation at 72 °C for 30 s. Data acquisition was performed at the end of each elongation step. The data were analysed using the manufacture's software with default settings for threshold values and baseline. The abundances of target molecules were calculated from the linear regression equation of the standard curve used in each qPCR run.

Supplementary Figure 2 | Experimental design and setup.

This setup was replicated for each microorganism exposure regime, e.g. non-toxic diatom *Thalassiosira weissflogii*, the PST producing dinoflagellate *Alexandrium minutum*, the pathogenic bacteria *Vibrio parahaemolyticus* together with *T. weissflogii*, and a combined treatment of *A. minutum* (100 cells/mL) and *V. parahaemolyticus* (10,000 cells/mL).

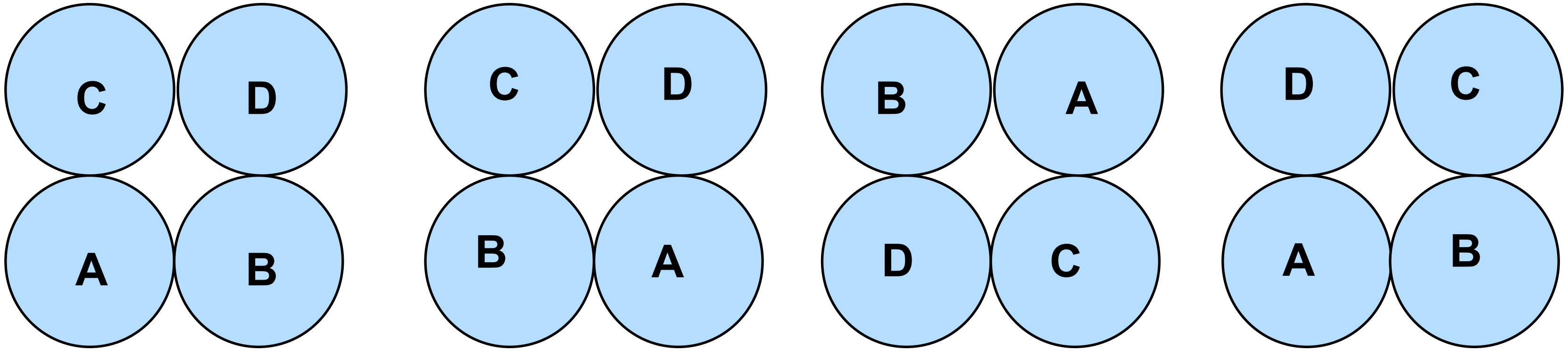

A = 28 °C/35 PSU (control)
B = 28 °C/31 PSU (hyposalinity)
C = 32 °C/35 PSU (warming)
D = 32 °C/31 PSU (warming + hyposalinity)

Each tank contained 16 mussels which were randomly assigned to four groups at the end of the exposure time:

Group one (four mussels):	i) Haemolymph was taken for lysosomal membrane stability<br>ii) Mantle tissue was taken for ATP, ADP, AMP, glucose and glycogen determination.<br>iii) Gill tissue was taken for determination of gill Na^+/K^+-ATPase activity.
Group two (four mussels):	Determination of metabolic rates (MO_2).
Group three (four mussels):	Toxin and/or bacterial concentration quantification.
Group four (four mussels):	Not analysed.

References

1 Guillard, R. R. L. Culture of phytoplankton for feeding marine invertebrates. In *Culture of Marine Invertebrate Animals* (eds W. L. Smith & M. H. Chanley) (Plenum Press, New York, 1975).

2 Raghunath, P. Virulence genes of seafood associated Vibrio parahaemolyticus. PhD thesis, Manipal University, Karnataka, India (2008).

3 Martinez-Gomez, C., Benedicto, J., Campillo, J. A. & Moore, M. Application and evaluation of the neutral red retention (NRR) assay for lysosomal stability in mussel populations along the Iberian Mediterranean coast. *J. Environ. Monitor.* **10**, 490-499 (2008).

4 Buratti, S. *et al.* Bioaccumulation of algal toxins and changes in physiological parameters in Mediterranean mussels from the North Adriatic Sea (Italy). *Environ. Toxicol.* **28**, 451-470 (2013).

5 Moore, M. N., Kohler, A., Lowe, D. & Viarengo, A. Lysosomes and autophagy in aquatic animals. In *Autophagy: Lower Eukaryotes and Non-Mammalian Systems, Pt A Methods in Enzymology* (ed D. J. Klionsky) (Elsevier Academic Press Inc, San Diego, 2008).

6 McCormick, S. D. Methods for nonlethal gill biopsy and measurement of Na^+, K^+-ATPase activity. *Can. J. Fish. Aquat. Sci.* **50**, 656-658 (1993).

7 Bergmeyer, H. *Methods of enzymatic analysis. Vol 6, Metabolites 1: Carbohydrates*. (VCH Verlagsgesellschaft, 1985).

8 Bergmeyer, H. *Methods of enzymatic analysis. Vol 7, Metabolites 2: Tri and dicarboxylic acids, purine, pyrimidines, inorganic compounds*. (VCH Verlagsgesellschaft, 1985).

9 Boundy, M. J., Selwood, A. I., Harwood, D. T., McNabb, P. S. & Turner, A. D. Development of a sensitive and selective liquid chromatography-mass spectrometry method for high throughput analysis of paralytic shellfish toxins using graphitic carbon SPE. *J. Chromatogr. A* **1387**, 1-12 (2015).

10 Turner, A. D., McNabb, P. S., Harwood, D. T., Selwood, A. I. & Boundy, M. J. Single-laboratory validation of a multitoxin ultra-performance LC-hydrophilic interaction LC-MS/MS method for quantitation of paralytic shellfish toxins in bivalve shellfish. *J. AOAC Int.* **98**, 609-621 (2015).

11 European Food Safety Authority. Scientific opinion of the panel on contaminants in the food chain on a request from the European Commission on marine biotoxins in shellfish - saxitoxin group. *EFSA Journal* **1019**,1-76 (2009).

12 Ausubel, F. M. *et al. Current Protocols in Molecular Biology*. (John Wiley & Sons Inc., New York, 1987).

13 Tyagi, A., Saravanan, V., Karunasagar, I. & Karunasagar, I. Detection of *Vibrio parahaemolyticus* in tropical shellfish by SYBR green real-time PCR and evaluation of three enrichment media. *Int. J. Food Microbiol.* **129**, 124–130 (2009).

14 Raghunath, P., Acharya, S., Blianumathi, A., Karunasagar, I. & Karunasagar, I. Detection and molecular characterization of *Vibrio parahaemolyticus* isolated from seafood harvested along the southwest coast of India. *Food Microbiol.* **25**, 824-830 (2008).
